# Small conductance Ca^2+^-activated K^+^ channels induce the firing pause periods during the activation of *Drosophila* nociceptive neurons

**DOI:** 10.7554/eLife.29754

**Published:** 2017-10-16

**Authors:** Koun Onodera, Shumpei Baba, Akira Murakami, Tadashi Uemura, Tadao Usui

**Affiliations:** 1 Graduate School of BiostudiesKyoto UniversityKyotoJapan; 2 Faculty of ScienceKyoto UniversityKyotoJapan; 3 Graduate School of InformaticsKyoto UniversityKyotoJapan; National Centre for Biological Sciences, Tata Institute of Fundamental ResearchIndia

**Keywords:** nociception, nocifensive behavior, calcium transient, potassium channel, chloride channel, *D. melanogaster*

## Abstract

In *Drosophila* larvae, Class IV sensory neurons respond to noxious thermal stimuli and provoke heat avoidance behavior. Previously, we showed that the activated neurons displayed characteristic fluctuations of firing rates, which consisted of repetitive high-frequency spike trains and subsequent pause periods, and we proposed that the firing rate fluctuations enhanced the heat avoidance (Terada et al., 2016). Here, we further substantiate this idea by showing that the pause periods and the frequency of fluctuations are regulated by small conductance Ca^2+^-activated K^+^ (SK) channels, and the *SK* knockdown larvae display faster heat avoidance than control larvae. The regulatory mechanism of the fluctuations in the Class IV neurons resembles that in mammalian Purkinje cells, which display complex spikes. Furthermore, our results suggest that such fluctuation coding in Class IV neurons is required to convert noxious thermal inputs into effective stereotyped behavior as well as general rate coding.

## Introduction

Animals sense diverse environmental inputs, including noxious ones, by using specific sensory organs. In principle, sensory neurons convert the intensity of stimuli into the magnitude of firing rates upon sensory transduction (Adrian, 1926). For instance, mammalian C-fiber nociceptors convert gentle touch stimuli into relatively low firing rates, whereas injurious forces elicit higher rates (Delmas et al., 2011). The ‘rate coding’ is valuable for sensory transduction, particularly with regard to stimulus intensity; however, the firing rate has an intrinsic upper limit because interspike intervals (ISIs) cannot be shorter than refractory periods, when the membrane is unable to respond to another stimulus (Berry and Meister, 1998; Hodgkin and Huxley, 1952). This implies that firing rates should saturate at high intensities, at which point the sensory inputs are no longer converted properly in an intensity-to-firing rate correspondence. Therefore, we assume that some sensory neurons may use other coding mechanisms that are employed in the central nervous system (Avissar et al., 2013; Haddad et al., 2013).

In *Drosophila* larvae, Class IV dendritic arborization neurons (Class IV neurons) are primary nociceptive neurons that respond to multiple stimuli, including high temperature, strong mechanical force, and short-wavelength light (Hwang et al., 2007; Tracey et al., 2003; Xiang et al., 2010). When the neurons are activated by noxious thermal stimuli, for instance, their sensory transduction provokes heat avoidance behavior where larvae rotate around the long body axis in a corkscrew-like manner. A large number of genes responsible for the neuronal activation were identified by evaluating behavioral phenotypes and monitoring Ca^2+^ dynamics in mutant strains (Lee et al., 2005; Neely et al., 2011; Tracey et al., 2003; Zhong et al., 2012); however, there have been few studies which have investigated the coding mechanism of the nociception by recording electrical activity (Terada et al., 2016; Xiang et al., 2010).

Previously, we built a measurement system using a 1460 nm infrared (IR) laser as a local heating device (Figure 1—figure supplement 1A) and found that Class IV neurons responded to noxious thermal stimuli with evoked characteristic fluctuations of firing rates, which consisted of repetitive high-frequency spike trains and subsequent quiescent periods (Terada et al., 2016). The occurrence of such ‘burst-and-pause’ firing patterns was coordinated with large Ca^2+^ increments over the entire dendritic arbors (designated as dendritic Ca^2+^ transients here) and was mediated by L-type voltage-gated Ca^2+^ channels (VGCCs). Knocking down L-type VGCCs in neurons abolished the burst-and-pause firing patterns, and the knockdown larvae displayed delayed heat avoidance behavior. Therefore, we hypothesized that the burst-and-pause firing patterns should be output signals transducing high intensity stimuli and provoking the robust avoidance behavior. However, the regulatory mechanism of the firing patterns remained unclear because L-type VGCCs produce depolarizing currents but not hyperpolarizing ones, which should underlie ‘pause’ periods. Here, we show that the pause period and the number of the burst-and-pause firing patterns are regulated by small conductance Ca^2+^-activated K^+^ (SK) channels, and that *SK* knockdown larvae display relatively fast heat avoidance. Furthermore, we show that one of the downstream neurons dramatically changes the response to two optogenetic activations of the Class IV neurons which have distinct numbers of burst-and-pause firing patterns. These findings strengthen the hypothesis and suggest that the ‘fluctuation coding’ is required to convert high intensities of noxious thermal stimuli into the robust, appropriate avoidance behavior as well as general rate coding.

## Results

### Dendritic Ca^2+^ transients precede unconventional spikes

**Figure 1.**
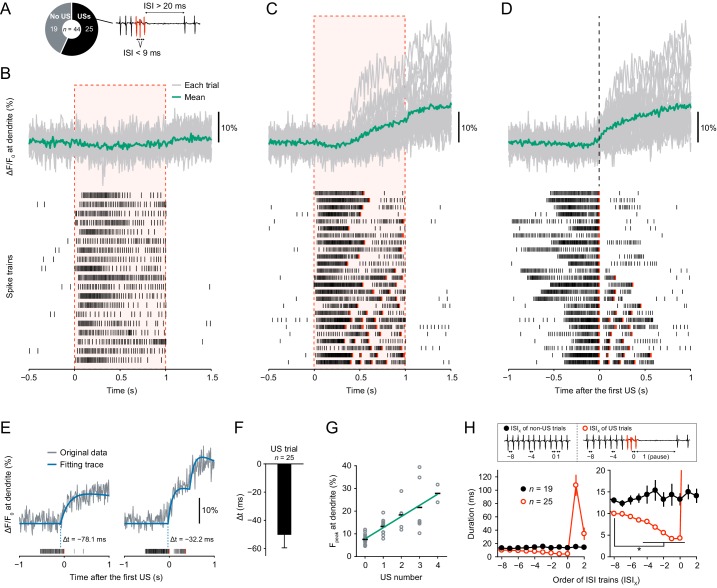
Dendritic Ca^2+^ transients precede unconventional spikes. Dual recordings of Ca^2+^ dynamics and extracellular membrane potential in Class IV neurons expressing GCaMP5G. A 44 mW IR laser was focused onto the proximal dendritic arbors in filet preparations for 1 s (red-dashed boxes in B and C). (**A**) Pie chart of recordings (total *n* = 44 cells). To the right, the example illustrates the definition of unconventional spikes (USs, an index of the burst-and-pause firing patterns) as follows: (i) The first and second ISIs of four sequential spikes are less than 9 ms. (ii) The third ISI is longer than 20 ms. Here, sets of the three spikes except for the last one are designated as USs. (**B–D**) Time courses of Ca^2+^ levels at distal dendrites (top) and spike trains (bottom). Data are classified into trials without USs (**B**) and with USs (**C–D**). Gray lines indicate dendritic Ca^2+^ transients from each cell, and the green line represents the averaged amplitude. Red raster lines indicate USs. (**B**) Trials without USs did not generate Ca^2+^ transients (*n* = 19 cells; ΔF/F_0_ = 0.52 ± 0.87%, mean ± s.e.m. after laser irradiation). (**C**) Trials with USs generated Ca^2+^ transients (*n* = 25 cells; ΔF/F_0_ = 9.33 ± 1.57%, mean ± s.e.m. after laser irradiation). The first USs occurred at 0.55 ± 0.04 s (mean ± s.e.m.). (D)Data (**C**) are aligned at the first-US end timings. The onset of the increase in Ca^2+^ levels approximately coincided with the first-US timings. (E)Representative time course of Ca^2+^ transients (gray) and the fitting traces (blue). The onset of Ca^2+^ transients actually preceded the first-US timings, and the Ca^2+^ transients with multiple USs were stepwise (right). (**F**) Temporal differences between the onset of Ca^2+^ transients and the first-US timings. The former occurred earlier than the latter (Δt = − 50.3 ± 9.2 ms, mean ± s.e.m.). (**G**) Amplitudes of F_peak_ are plotted against total US numbers for each trial. Short black bars indicate the averages of F_peak_, and the green line is a linear regression of plotted data (p=1.0 × 10^−11^, *rho* = 0.82, Spearman’s rank correlation test). (**H**) Time course of ISIs. X of ISI_X_ indicates the order of ISIs: (black) ISI_−8_–ISI_2_ are the minimum ISI trains of non-US trials in Figure 1B. (red) ISI_0_ indicates the ISIs of the first-US end, and ISI_1_ represents the pause periods in Figure 1C. At the right, the y-axis was magnified to show that ISIs became shorter before the occurrence of the pause (mean ± s.e.m.; *p<0.05, paired-sample *t*-test with Bonferroni correction). 10.7554/eLife.29754.004Figure 1—source data 1.Source data for Figure 1. 10.7554/eLife.29754.005Figure 1—source data 2.Source data for Figure 1—figure supplement 1.

To understand the molecular mechanism that generates burst-and-pause firing patterns in response to thermal stimuli, we first examined the temporal relationship with dendritic Ca^2+^ transients, whose occurrence was coordinated with the specific firing patterns in an all-or-none fashion. The temporal relationship between the Ca^2+^ transients and unconventional spikes (USs; Figure 1A) was unclear because the temporal resolution of monitoring Ca^2+^ dynamics was 30 Hz in our previous work (Terada et al., 2016), which was lower than the minimum frequency required to measure differences between spike timings (100–500 Hz; Lütcke et al., 2013). Therefore, we monitored Ca^2+^ dynamics with higher temporal resolution (100 Hz) by using a genetically encoded Ca^2+^ indicator GCaMP5G (Akerboom et al., 2012), which was brighter than the ratiometric indicator TN-XXL (Mank et al., 2008) as employed in our previous work (Terada et al., 2016). We then found that all the dendritic Ca^2+^ transients occurred concurrently with USs (Figure 1B,C and D). In contrast, when no US occurred or before the first US occurred, Ca^2+^ transients were never observed. To accurately measure the onset of Ca^2+^ transients with stochastic fluctuations, we used an event detection algorithm based on a Schmitt trigger approach (Grewe et al., 2010; Lütcke et al., 2013) and fit each transient to exponential curves. We then found that the onset of Ca^2+^ transients preceded first-US timings and that the Ca^2+^ transients with multiple USs were stepwise (Figure 1E and F; Δt = − 50.3 ± 9.2 ms, mean ± s.e.m.). We also found that the peak amplitudes of Ca^2+^ transients displayed a positive linear correlation with the number of USs (Figure 1G; p=1.0 × 10^−11^, *rho* = 0.82, Spearman’s rank correlation test). Furthermore, ISIs were shorter before onsets of pauses (Figure 1H; p<0.05, paired-sample *t*-test with Bonferroni correction).

**Figure 1—figure supplement 1.**
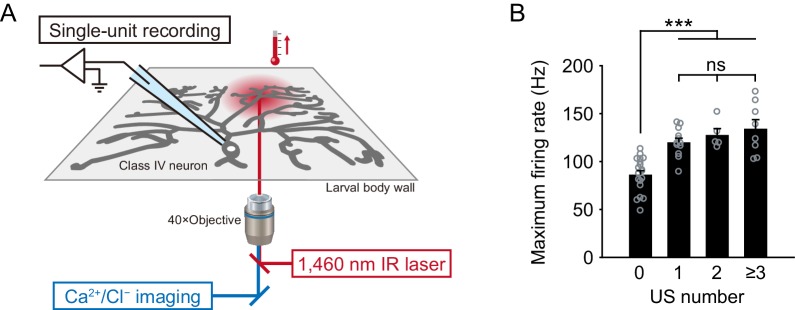
The recording system and maximum firing rates regarding the US number. (**A**) A schematic diagram of the recording system. As a heating device, the IR-laser beam passes through an objective and targets Class IV neurons in whole- or filet-mounted larvae. We measured firing responses of the neuronal somata using an extracellular recording electrode in filet preparations, and acquired Ca^2+^ or Cl^−^ dynamics in whole-mount or filet preparations. (**B**) Maximum firing rates from Figure 1B–C regarding the US number. The maximum firing rates of US trials (Figure 1C) was larger than that of non-US ones (Figure 1B; mean ± s.e.m.; ***p<0.001, Student’s *t*-test with Bonferroni correction), but it did not significantly change among trials with varying numbers of US.

We hypothesized that the Ca^2+^ influx mediated by L-type VGCCs amplifies membrane depolarization, which narrows down ISIs of bursts and also induces subsequent pauses. Therefore, we searched for ion channels responsible for generating inhibitory currents that could hyperpolarize membrane potentials during pause periods. We anticipated that activities of such channels must be regulated by intracellular Ca^2+^ concentration ([Ca^2+^]_i_) either directly or indirectly.

### Electrophysiological screen of Cl^−^ channels and K^+^ channels

**Figure 2.**
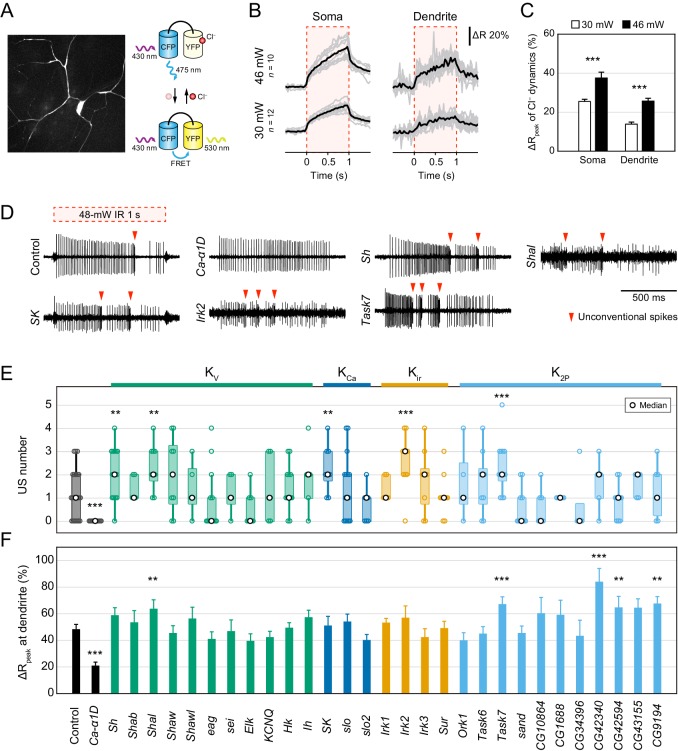
Electrophysiological screen of Cl^−^ and K^+^ channels. (**A–C**) Cl^−^ dynamics of Class IV neurons expressing SuperClomeleon. The IR laser (30 and 46 mW) was focused onto the proximal dendritic arbors in whole-mount preparations for 1 s (red-dashed boxes in B). (**A**) A schematic diagram of Cl^−^ indicator SuperClomeleon. The FRET ratio decreases upon an influx of Cl^−^, due to quenching of YFP fluorescence by reversible Cl^−^ binding. Left is a representative CFP image before IR-laser irradiation. (**B**) Time courses of the FRET ratio at somata (left) and distal dendrites (right) in wild-type neurons. Both of them increased upon IR-laser irradiation. Gray lines indicate each of the Cl^−^ changes, and black lines represent the averaged amplitudes. The apparent efflux of Cl^−^ ions was unexpected. (**C**) Amplitudes of ΔR_peak_ of SuperClomeleon increased with IR-laser power (mean ± s.e.m.; ***p<0.001, Student’s *t*-test). (**D–F**) Responses of screened neurons expressing the Ca^2+^ indicator TN-XXL. The 48 mW IR laser was focused onto the proximal dendritic arbors in filet preparations for 1 s (red-dashed box in D). **p<0.05, ***p<0.01 versus control. (**D**) Representative recordings of control, *Ca-α1D* (L-type VGCC α_1_ subunit gene) RNAi and K^+^ channel-coding gene (*Shaker*, *Shal*, *SK*, *Irk2* and *Task7*) RNAi neurons. (**E**) Boxplot of the total US number in screened neurons. The US number increased in five different gene knockdown neurons (*Sh*, *Shal*, *SK*, *Irk2* and *Task7*; Wilcoxon rank sum test). (**F**) Amplitudes of the dendritic Ca^2+^ transients in screened channels. The amplitudes did not decrease except for *Ca-α1D* RNAi neurons (mean ± s.e.m.; Student’s *t*-test). Bottom horizontal labels indicate symbols of knocked down genes and upper labels represent channel families: K_v_, voltage-gated K^+^ channel; K_Ca_, Ca^2+^-activated K^+^ channel; K_ir_, Inward rectifier K^+^ channel; K_2P_, Two-pore domain K^+^ channel. 10.7554/eLife.29754.009Figure 2—source data 1.Twenty-Nine K^+^ channels were screened.*w* was a negative control and *Ca-α1D* was a positive control whose knockdown abolished burst-and-pause firing patterns. 10.7554/eLife.29754.010Figure 2—source data 2.Source data for Figure 2. 10.7554/eLife.29754.011Figure 2—source data 3.Source data for Figure 2—figure supplement 1. 10.7554/eLife.29754.012Figure 2—source data 4.Source data for Figure 2—figure supplement 2.

To elucidate the regulatory mechanism of the pause, we screened ion channels that might generate hyperpolarizing currents: such channels include outward K^+^ and inward Cl^−^ channels. The direction of passive transport of each ion through channels is dependent on the electrochemical gradient across the plasma membrane, but the intracellular Cl^−^ concentration ([Cl^−^]_i_) is largely different among cells (Kaila et al., 2014) and the direction of Cl^−^ transport in Class IV neurons was unclear. We therefore monitored Cl^−^ dynamics by a genetically encoded FRET-based Cl^−^ indicator, SuperClomeleon (Grimley et al., 2013; Figure 2A) and found that the FRET ratio increased at both somata and distal dendrites upon IR-laser irradiation (Figure 2B and C). Because the FRET ratio of SuperClomeleon rises as the [Cl^−^]_i_ falls, we expected that the [Cl^−^]_i_ should decrease upon stimulation. These results suggest that the passive Cl^−^ transport in Class IV neurons is outward and generates depolarizing currents but not hyperpolarizing ones. In parallel, we investigated the role of one of the Ca^2+^-activated Cl^−^ channels, Subdued (Jang et al., 2015), and showed that it can contribute to membrane excitation in the neurons (Figure 2—figure supplement 1). Thus, we excluded Cl^−^ channels as candidate sources of hyperpolarizing currents.

**Figure 2—figure supplement 1.**
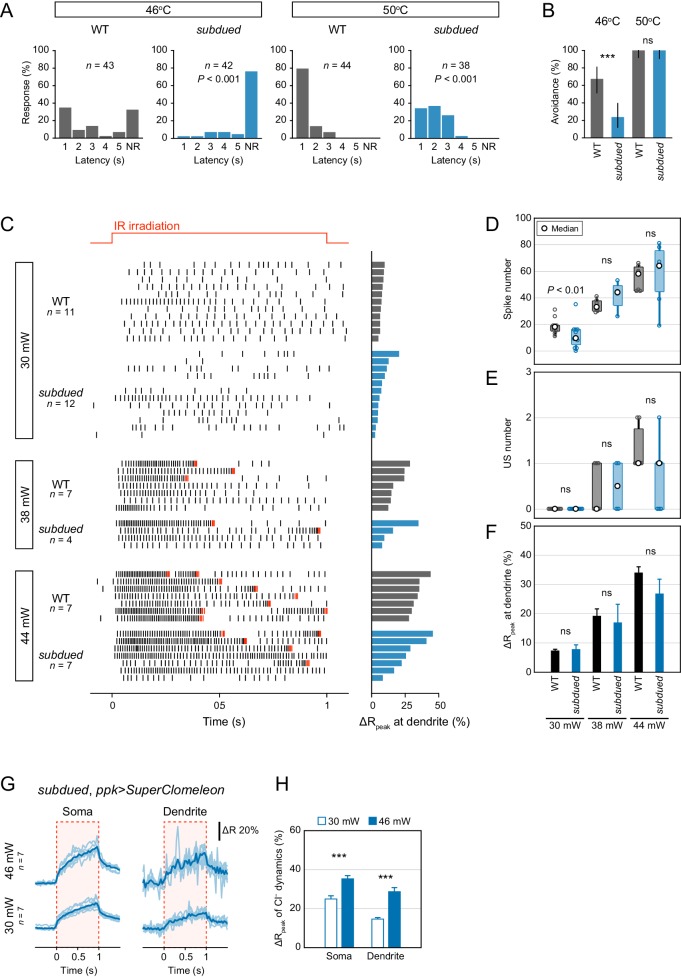
Behavioral and electrophysiological analysis of *subdued* mutants. The gating property of subdued channels is dependent on heat, voltage and Ca^2+^, and the expression in Class IV neuron is required for larval heat avoidance behavior (Jang et al., 2015). Considering the results of the Cl^−^ ion direction (Figure 2B–C), the possibility of subdued channels contributing to inhibitory currents is low, although an effect of the Subdued channels on neuronal activity cannot be completely excluded. (see below). (**A–B**) Avoidance behavior of wild-type and *subdued* mutant larvae expressing TN-XXL in response to thermal stimulation (46°C and 50°C). The onsets of avoidance behavior were delayed at both temperatures, and the fraction of no response was increased in mutant larvae at 46°C. (**A**) The distribution of response latency (Wilcoxon rank sum test). NR, no response group. (**B**) Percentage of larvae responding within 5 s with 95% Clopper-Pearson confidence intervals (Fisher’s exact test). (**C–F**) Responses of wild-type and *subdued* mutant neurons expressing TN-XXL with different IR-laser power (30, 38 and 44 mW). The IR laser was focused onto the proximal dendritic arbors in filet preparations for 1 s (upper red line in C). (**C**) Raster plots of firing (left) and magnitudes of the ΔR_peak_ corresponding to dendritic Ca^2+^ transients (right). Trials are sorted by descending order of the magnitude of the ΔR_peak_. Red raster lines indicate USs. (**D**) Spike numbers decreased in *subdued* mutant neurons only at low IR-laser power (30 mW; boxplot; Student’s *t*-test). (**E–F**) *subdued* mutant neurons did not significantly change the number of USs (E; boxplot, Wilcoxon rank sum test) or the amplitudes of the dendritic Ca^2+^ transients (F; mean ± s.e.m., Student’s *t*-test). (**G–H**) Cl^−^ dynamics of *subdued* mutant neurons expressing SuperClomeleon. The IR laser (30 and 46 mW) was focused onto the proximal dendritic arbors in whole-mount preparations for 1 s (red-dashed boxes in G). (**G**) Time courses of the FRET ratio of SuperClomeleon at somata (left) and distal dendrites (right) in *subdued* mutant neurons. The ratio increased upon IR irradiation. Light blue lines indicate each of the Cl^−^ changes, and dark lines represent the averaged amplitudes. (**H**) Amplitudes of the ΔR_peak_ for SuperClomeleon increased with IR-laser power in *subdued* mutant neurons (mean ± s.e.m., Student’s *t*-test), but they were not significantly different from the values obtained for wild-type neurons in Figure 2C. Furthermore, there were no changes in physiological activities in *subdued* knockdown neurons (data not shown). These results suggested that Subdued channels can contribute to membrane excitation in Class IV neurons, although their function may be rather limited; in any case, the channels were not a source of inhibitory currents. *p<0.05, **p<0.01, ***p<0.001.

**Figure 2—figure supplement 2.**
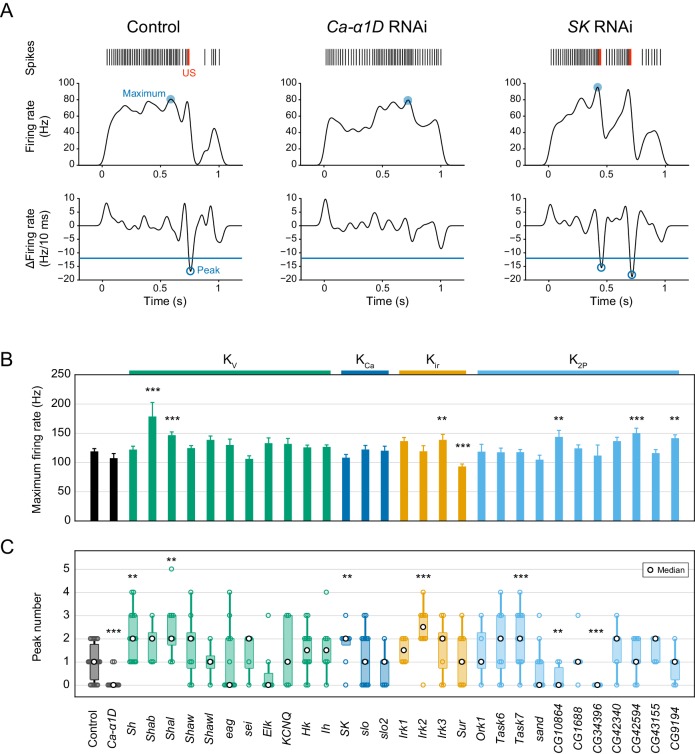
Quantification of the maximum firing rate and the peak number. (**A**) Representative traces of control, *Ca-α1D*, and *SK* RNAi neurons. Firing rates (middle) were computed from spike trains (top) by Gaussian kernel methods (σ = 25 ms; Shimazaki and Shinomoto, 2010). The temporal differences in the firing rate were calculated (bottom, per 10 ms), and peaks of the time derivative were specified below a threshold (−12 Hz/10 ms) where the firing rate decreased by 60 Hz for 50 ms. The local minimum was more closely matched to detect burst-and-pause firing patterns than the local maximum that was used in our previous work (Terada et al., 2016). (**B**) Bar plots of the maximum firing rates in screened neurons (mean ± s.e.m., Student’s *t*-test). (**C**) Boxplots of the peak numbers in screened neurons. The peak number increased in five different gene knockdown neurons (*Sh*, *Shal*, *SK*, *Irk2* and *Task7*; Wilcoxon rank sum test). Bottom horizontal labels indicate symbols of knocked down genes and upper labels indicate channel families: K_v_, voltage-gated K^+^ channel; K_Ca_, Ca^2+^-activated K^+^ channel; K_ir_, Inward rectifier K^+^ channel; K_2P_, Two-pore domain K^+^ channel. **p<0.05, ***p<0.01 versus control.

Next, we focused on the roles of various K^+^ channels as mediators of hyperpolarization. The *Drosophila melanogaster* genome has 29 genes that encode pore-forming subunits of K^+^ channels, including 11 voltage-gated K^+^ channels (*Sh*, *eag*, etc.), 11 two-pore domain K^+^ channels (*Task6*, *sand*, etc.; Pimentel et al., 2016) and others (Figure 2—source data 1). We first knocked down each of the candidate genes in Class IV neurons and recorded electrical activities of the knockdown neurons upon IR-laser irradiation, and examined the properties of burst-and-pause firing patterns. We found that the number of USs was significantly increased in five different gene knockdown neurons (*Sh*, *Shal*, *SK*, *Irk2,* and *Task7*; Figure 2D and E; p<0.05, Wilcoxon rank sum test). The five knockdown neurons also exhibited an increased number of ‘peaks’ (Figure 2—figure supplement 2; p<0.05, Wilcoxon rank sum test), as defined in our previous study (Terada et al., 2016). Furthermore, although knocking down L-type VGCC abolishes dendritic Ca^2+^ transients (Terada et al., 2016), the five K^+^ channel knockdowns did not decrease the amplitudes of the Ca^2+^ transients (Figure 2F). These results suggested that these candidates participate in an unknown mechanism downstream of the Ca^2+^ influx. Notably, *SK* encodes a small conductance Ca^2+^-activated K^+^ channel, which can be activated by dendritic Ca^2+^ influx, and therefore could be one of the major factors underlying hyperpolarization. Thus, we explored how SK channels contributed to shaping the burst-and-pause firing patterns.

### SK channels generate pause periods

**Figure 3.**
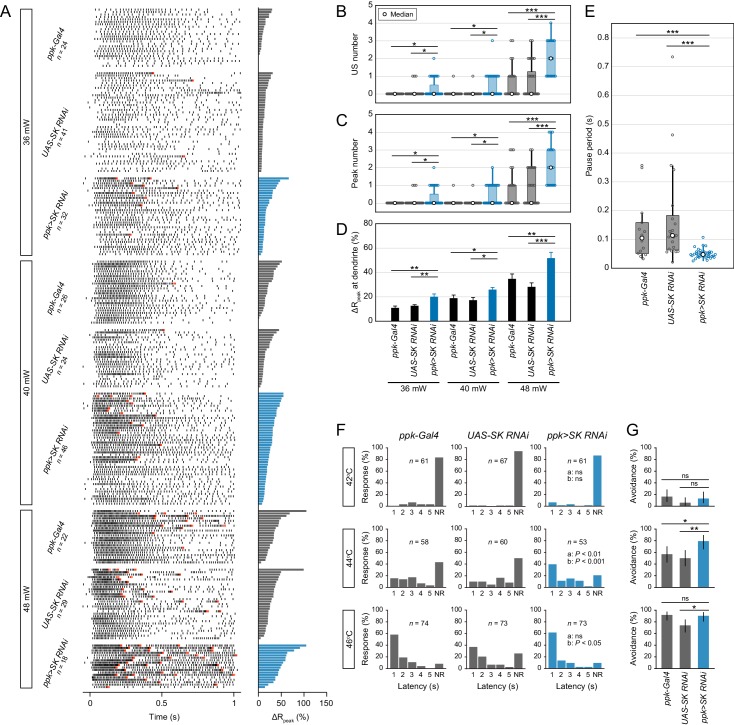
SK channels generate pause periods. (**A–E**) Responses of two control neurons (*ppk-Gal4* and *UAS-SK RNAi*^HMJ21196^) and *SK* knockdown neurons (*ppk>SK RNAi*^HMJ21196^) with different IR-laser power settings (36, 40 and 48 mW). The IR laser was focused onto the proximal dendritic arbors in filet preparations for 1 s. (A)Raster plots of firing (left) and magnitudes of the ΔR_peak_ corresponding to dendritic Ca^2+^ transients (right). Trials are sorted in descending order of the magnitude of the ΔR_peak_. Red raster lines indicate USs. (**B–D**) *SK* knockdown neurons increased the US number, peak number (B and C; boxplots; Wilcoxon rank sum test with Holm correction), and amplitude of the dendritic Ca^2+^ transients (D; mean ± s.e.m.; Student’s *t*-test with Holm correction) with three different laser powers. (**E**) Boxplots of the pause periods triggered by the 48 mW IR laser. Pause periods were shortened in *SK* knockdown neurons (median: [*ppk-Gal4*] 103.9 ms (*n* = 14), [*UAS-SK RNAi*] 112.9 ms (*n* = 21), [*ppk >SK RNAi*] 46.75 ms (*n* = 34); Student’s *t*-test with Holm correction). (**F–G**) Avoidance behavior of two control larvae and *SK* knockdown larvae in response to thermal stimulation (42, 44, and 46°C). (**F**) The distribution of response latency. *SK* knockdown larvae displayed fast onsets of responses upon moderate stimulation (44°C; median: [*ppk-Gal4*] 3.80 s, [*UAS-SK RNAi*] 4.86 s, [*ppk>SK RNAi*] 1.80 s; Wilcoxon rank sum test with Holm correction). Neither control nor *SK* knockdown larvae showed avoidance behavior upon lower stimulation (42°C), whereas most of the larvae displayed it with higher stimulation (46°C). NR, no response group. ‘a’ is a *P* value versus *ppk-Gal4*, and ‘b’ is that versus *UAS-SK RNAi*. (**G**) Percentage of larvae responding within 5 s with 95% Clopper-Pearson confidence intervals. The response rate of *SK* knockdown larvae increased upon moderate stimulation (44°C: [*ppk-Gal4*] 56.9%, [*UAS-SK RNAi*] 50.0%, [*ppk>SK RNAi*] 79.2%; Fisher’s exact test with Holm correction). *p<0.05, **p<0.01, ***p<0.001. 10.7554/eLife.29754.019Figure 3—source data 1.Source data for Figure 3. 10.7554/eLife.29754.020Figure 3—source data 2.Source data for Figure 3—figure supplement 1. 10.7554/eLife.29754.021Figure 3—source data 3.Source data for Figure 3—figure supplement 2. 10.7554/eLife.29754.022Figure 3—source data 4.Source data for Figure 3—figure supplement 3. 10.7554/eLife.29754.023Figure 3—source data 5.Source data for Figure 3—figure supplement 4. 10.7554/eLife.29754.024Figure 3—source data 6.Source data for Figure 3—figure supplement 5.

To investigate the physiological roles of SK channels, we stimulated the knockdown neurons with different IR-laser powers. We then found that *SK* knockdown increased the firing properties, including the US number, peak number and maximum firing rate, even with low laser powers (Figure 3A–D, Figure 3—figure supplement 1A). Importantly, the *SK* knockdown shortened the pause periods (Figure 3E; median of pause period: [*ppk-Gal4*] 103.9 ms, [*UAS-SK RNAi*] 112.9 ms, [*ppk>SK RNAi*] 46.75 ms; p<0.001, Student’s *t*-test with Holm correction), which suggested that SK-dependent current regulates the pause period. Similar firing changes were also observed in two additional *SK* knockdown neurons, targeting two different sequences in the *SK* gene (Figure 3—figure supplement 2A–D, Figure 3—figure supplement 1B). We speculated that SK channels would be dramatically activated by the sudden increase in [Ca^2+^]_i_ through L-type VGCCs, and would generate a transient hyperpolarizing K^+^ current. Nevertheless, it was important to rule out a more trivial explanation for the changes in firing patterns in the *SK* knockdown neurons, which might possibly be due to altered dendritic architecture. We quantified the dendritic morphology of *SK* knockdown neurons and concluded that the altered physiological responses were not due to morphological defects in the dendritic arbors (Figure 3—figure supplement 3).

**Figure 3—figure supplement 1.**
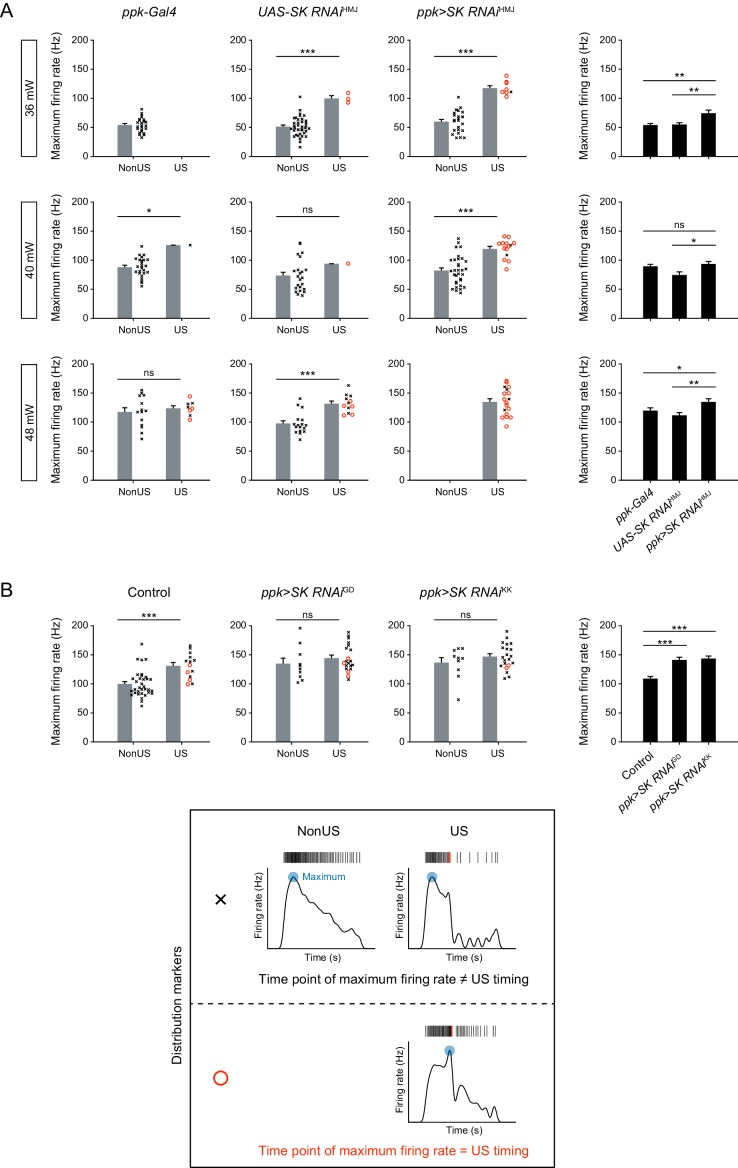
Maximum firing rates regarding the presence of USs and the effect of *SK* knockdown. (Left 3 columns) Data were classified into two groups: non-US and US. When USs occurred, the maximum firing rate tended to increase and several time points of the maximum firing rate overlapped with US timings. Gray bars indicate mean + s.e.m., and the right scatter plots show the distribution of all trials (see the bottom box). (Right 1 column) Maximum firing rate including both non-US and US trials. *SK* knockdown tended to increase the maximum firing rates upon all laser powers tested. Black bars indicate mean +s.e.m. (**A**) Quantification of Figure 3A. (**B**) Quantification of Figure 3—figure supplement 2A. *p<0.05, **p<0.01, ***p<0.001, Student’s *t*-test with Holm correction.

**Figure 3—figure supplement 2.**
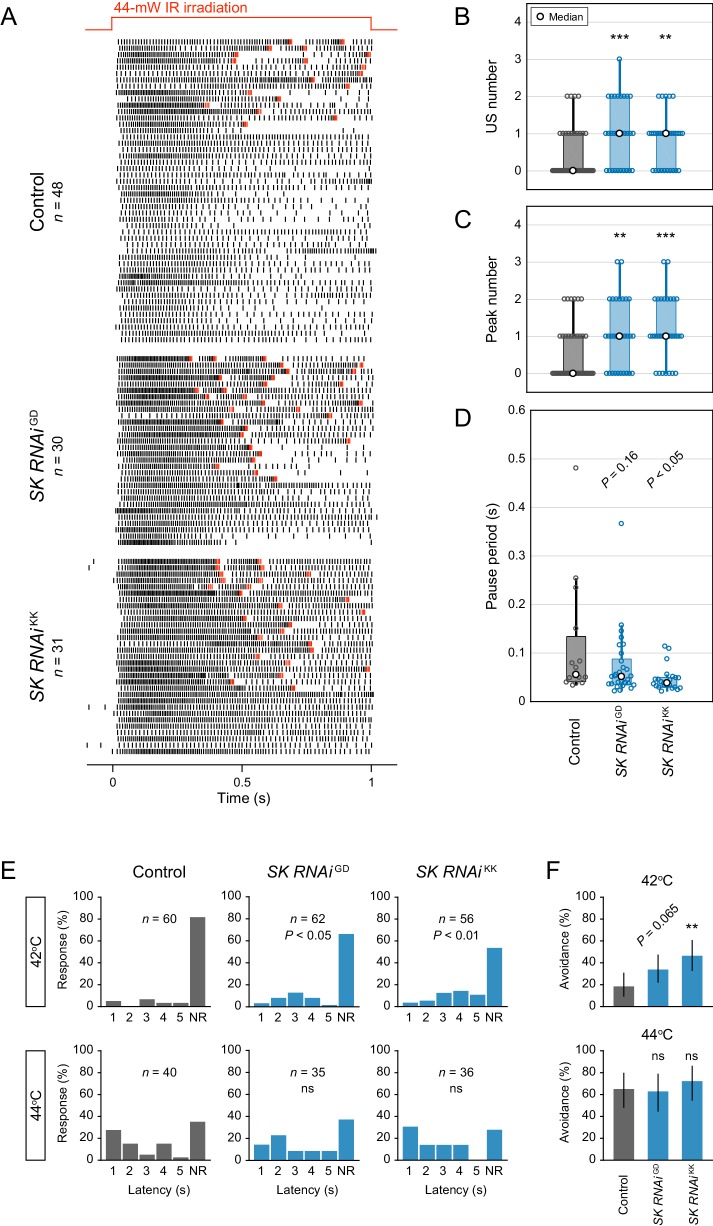
Analysis of two different *SK* knockdowns. (**A–D**) Responses of control neurons and two different *SK* knockdown neurons (*ppk>SK RNAi*^GD12601^, *ppk>SK RNAi*^KK107699^) with different target sequences. The 44 mW IR laser was focused onto the proximal dendritic arbors in filet preparations for 1 s (upper red line in A). (**A**) Raster plots of firing. Red raster lines indicate USs. (**B and C**) Both *SK* knockdowns increased the US number and peak number in Class IV neurons (Wilcoxon rank sum test with Holm correction). (**D**) Boxplots of the pause periods. Pause periods tended to decrease in the *SK* knockdown neurons (median: [Control] 55.3 ms (*n* = 15), [*SK RNAi*^GD^] 51.1 ms (*n* = 29), and [*SK RNAi*^KK^] 38.1 ms (*n* = 24); Student’s *t*-test with Holm correction). (**E–F**) Avoidance behavior of control and the two *SK* knockdown larvae in response to thermal stimulation (42°C and 44°C). The *SK* knockdown larvae displayed faster onsets of responses, and the frequency of responses tended to increase compared to the control (42°C). (**E**) The distribution of response latency (Wilcoxon rank sum test with Holm correction). NR, no response group. (**F**) Percentage of larvae responding within 5 s with 95% Clopper-Pearson confidence intervals (Fisher’s exact test with Holm correction). The pause periods were not completely abolished upon *SK* knockdown (see also Figure 3E), which raised the possibility that other Ca^2+^-activated K^+^ channels (e.g., *slowpoke* and *slo2*) could compensate for the loss of SK function. However, the pause periods of *SK* knockdown neurons did not decrease further by adding charybdotoxin (1 µM; K_Ca_ blocker; data not shown). *p<0.05, **p<0.01, ***p<0.001.

**Figure 3—figure supplement 3.**
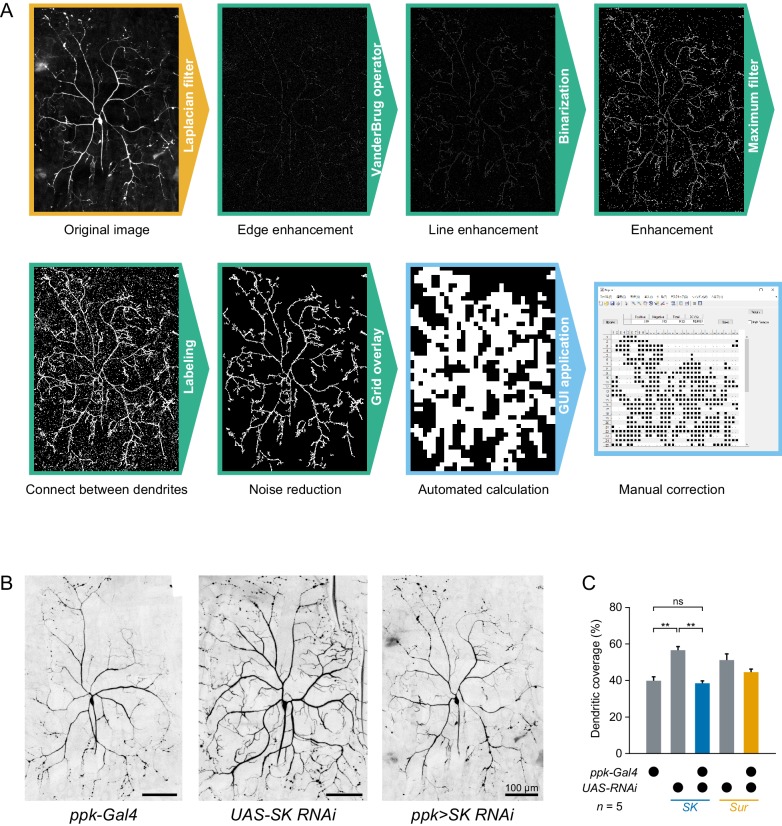
Dendritic morphology of *SK* knockdown Class IV neurons. It was recently reported that heat avoidance behavior is impaired in neuropeptide knockdown larvae, which show defects in the dendritic morphology of Class IV neurons (Honjo et al., 2016). This observation raised the possibility that such morphological defects might affect electrophysiological responses of the neurons. Therefore, we examined whether the *SK* knockdown also caused gross morphological defects in the neurons or not. To quantify dendritic morphology, we measured dendritic coverage (Honjo et al., 2016) with modifications. (A)Image processing for measuring dendritic coverage (see also Materials and methods). (B)Representative images of two control neurons and *SK* knockdown neurons. Here black and white in the original images are inverted to facilitate visualization. (C)Quantification of dendritic coverage ([*ppk-Gal4*] 39.8 ± 2.2%, [*UAS-SK RNAi*] 56.6 ± 2.0%, [*ppk>SK RNAi*] 38.4 ± 1.3%, mean ± s.e.m.; **p<0.01, Student’s *t*-test with Holm correction). Dendritic coverage of *SK* knockdown neurons did not increase compared to those of the two controls. These results indicate that the physiological alterations of *SK* knockdown neurons are not simply due to morphological defects of dendritic arbors, but are a direct consequence of the different composition of K^+^ channels. Although the dendritic coverage of *UAS-SK RNAi* was relatively higher than *ppk-Gal4* and *ppk>SK RNAi*, the difference may be explained by the observation that the coverage of other transgenic neurons were different in the absence or presence of *ppk-Gal4* drivers ([*UAS-Sur RNAi*] 51.2 ± 3.4%, [*ppk>Sur RNAi*] 44.6 ± 1.6%, mean ± s.e.m.).

**Figure 3—figure supplement 4.**
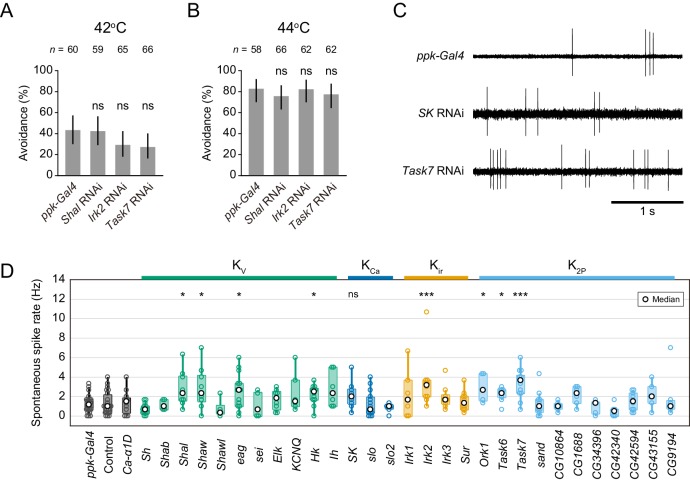
Avoidance behavior and spontaneous spikes upon knockdown of the other K^+^ channels. (**A–B**) Avoidance behavior of driver control larvae (*ppk-Gal4*) and the knockdown larvae of three K^+^ channels (*Shal*, *Irk2*, and *Task7* RNAi) in response to thermal stimulation (A: 42°C, B: 44°C). Percentage of larvae responding within 5 s did not change significantly in the knockdown larvae (Fisher’s exact test). Black bars indicate 95% Clopper-Pearson confidence intervals. We could not investigate the behavior of *Sh* knockdown larvae because they hardly developed into mature larvae. (**C**) Representative recordings of spontaneous spikes in knockdown Class IV neurons. (**D**) Spontaneous spike rate in knockdown neurons. The rates were quantified from the spike trains for 3 s before stimulation ([*ppk-Gal4*] *n* = 22, [others] see Figure 2—source data 1). The rates increased upon *Shal*, *Irk2* and *Task7* knockdowns; however, that of *SK* knockdown did not change significantly (Wilcoxon rank sum test compared to *ppk-Gal4* driver control). Bottom horizontal labels indicate symbols of knocked down genes and upper labels represent channel families: K_v_, voltage-gated K^+^ channel; K_Ca_, Ca^2+^-activated K^+^ channel; K_ir_, Inward rectifier K^+^ channel; K_2P_, Two-pore domain K^+^ channel. *p<0.05, **p<0.01, ***p<0.001.

**Figure 3—figure supplement 5.**
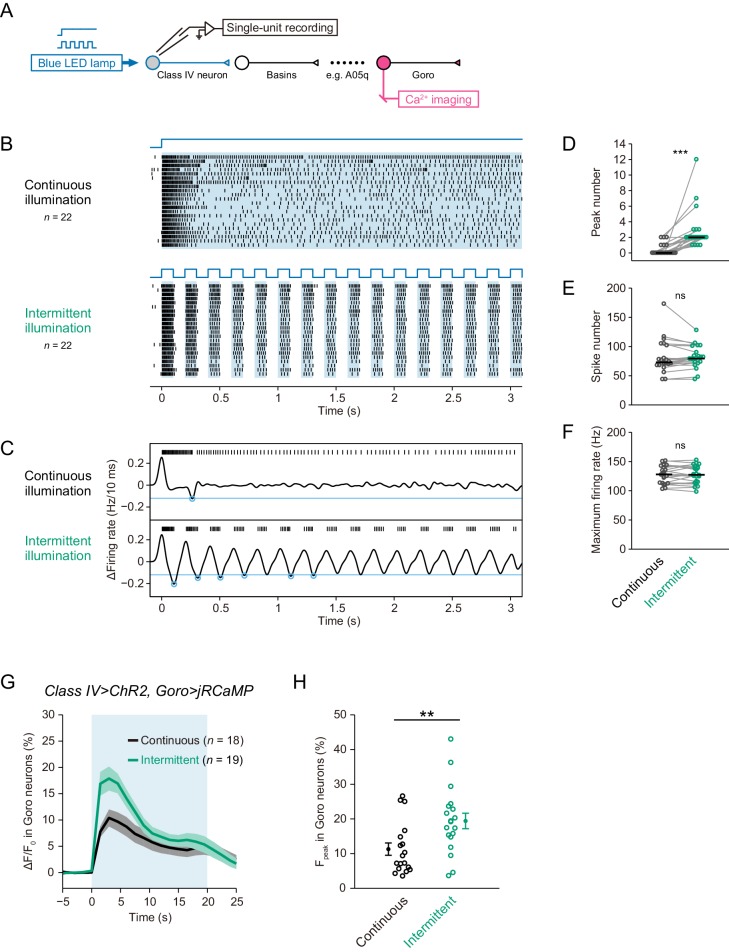
Differential Ca^2+^ dynamics of downstream neurons induced by optogenetic activations of Class IV neurons. (**A**) A schematic diagram of the recording system. We activated ChR2-expressing Class IV neurons by blue LED illumination (0.37 mW/mm^2^) and measured the firing responses by extracellular recording in filet preparations. We also acquired Ca^2+^ dynamics of jRCaMP-expressing downstream neurons in optimized preparations. (**B–F**) Responses of Class IV neurons activated with a blue LED for 3 s in a continuous fashion (‘continuous illumination’) or in a pulsatile one (‘intermittent illumination’). Each cell was stimulated by two illumination patterns temporally separated by a short resting interval of at least 1.5 min. (B)Raster plots of firing. Upper blue lines indicate the time course of LED illumination. (**C**) Representative traces of spike trains and the time derivative. Blue circles indicate negative peaks of the time derivative below a threshold (blue line, −12 Hz/10 ms). Even continuous illumination induced one or two peaks in some trials possible because of endogenous L-type VGCCs, but the number was much less than that upon intermittent illumination. (**D–F**) Intermittent illumination induced more peak number in Class IV neurons than continuous illumination did (D; Wilcoxon signed-rank sum test); however, the two illumination patterns did not change total spike number (E; Wilcoxon signed-rank sum test) and maximum firing rate (F; Paired-sample *t*-test). Black short bars indicate median. (**G–H**) Ca^2+^ dynamics of jRCaMP-expressing Goro neurons induced by optogenetic activations of Class IV neurons with a blue LED for 20 s (blue shade in G). Each sample was illuminated with a continuous or intermittent pattern. (**G**) Time courses of Ca^2+^ levels at soma (mean ± s.e.m.). The fluorescence rapidly increased within the first 3 s upon both illuminations. (H)Maximum amplitudes of Ca^2+^ rises were larger upon intermittent activation than a continuous one (Welch’s *t*-test). Dots and bars are mean ± s.e.m. *p<0.05, **p<0.01, ***p<0.001.

We hypothesized that the burst-and-pause firing patterns should be output signals provoking robust heat avoidance behavior. To test this hypothesis, we examined how *SK* knockdown larvae responded to thermal stimulation, and we found that they displayed significantly faster onsets of responses; moreover, the response rate was increased upon moderate stimulation (44°C, Figure 3F and G; 42°C, Figure 3—figure supplement 2E F). These results suggested that the enhanced behavioral responses are induced either by the increment in the US number or by the changes in the other firing properties (pause period and maximum firing rate, etc.). We previously reported that the L-type VGCC knockdown abolished the burst-and-pause firing patterns and provoked a delayed response to thermal stimuli (Terada et al., 2016). Consistent with these findings, we propose that the burst-and-pause firing patterns should be output signals provoking the robust avoidance behavior.

We also examined heat avoidance behavior of larvae, where one of the other three candidate genes (*Shal*, *Irk2*, and *Task7*) was knocked down; however, the respective knockdown larvae did not show any difference in the response rate of avoidance (Figure 3—figure supplement 4A and B). Interestingly, the frequencies of spontaneous spikes were significantly increased in the three knockdown Class IV neurons but not in *SK* knockdown ones (Figure 3—figure supplement 4C and D). Notably, a recent study revealed that the activation of Class IV neurons during larval development inhibited the synaptic transmission to second-order neurons via serotonergic feedback signaling and suppressed the avoidance behavior (Kaneko et al., 2017). In addition, the topographic projections of Class IV neurons are partially dependent on the levels of neuronal activity, including spontaneous spikes (Kaneko and Ye, 2015; Yang et al., 2014). Thus, the elevated basal neuronal activity during development might decrease the efficacy of synaptic transmission and/or remodel synaptic connections of the neurons, which would tend to counteract the effect of the increment of firing rate fluctuations on the avoidance behavior of these knockdown animals.

The downstream circuitry of the Class IV neurons has been identified through functional and anatomical approaches (Chin and Tracey, 2017; Hu et al., 2017; Ohyama et al., 2015; Vogelstein et al., 2014; Yoshino et al., 2017). To explore any differences in the responses of that circuitry when Class IV neurons evoked various firing patterns, we examined the neuronal activity of Goro neurons in response to optogenetic activations of Class IV neurons (Figure 3—figure supplement 5A). We first investigated firing patterns induced by optogenetic activations in Class IV neurons and found that the numbers of burst-and-pause patterns were significantly different between continuous and intermittent illuminations (Figure 3—figure supplement 5B–D; p<0.001, Wilcoxon signed-rank sum test). Importantly, the total spike numbers and maximum firing rates were comparable between the two conditions (Figure 3—figure supplement 5E and F). We then examined whether the activity of the downstream neurons should be differentially induced with the type of optogenetic manipulation. We found that the maximum amplitude of Ca^2+^ rises in Goro neurons was larger upon activation accompanied with more burst-and-pause firing patterns in Class IV neurons (Figure 3—figure supplement 5G and H; [continuous] F_peak_ = 11.3 ± 1.8%, [intermittent] F_peak_ = 19.4 ± 2.2%, mean ± s.e.m.; p<0.01, Welch’s *t*-test). The results suggested that firing rate fluctuations were decoded in downstream circuits separately from the firing rate itself. Although the mechanism by which burst-and-pause firing patterns are read out as downstream electrical signals is still unknown, we can address this question by examining the activities of the other neurons in the circuitry.

## Discussion

**Figure 4.**
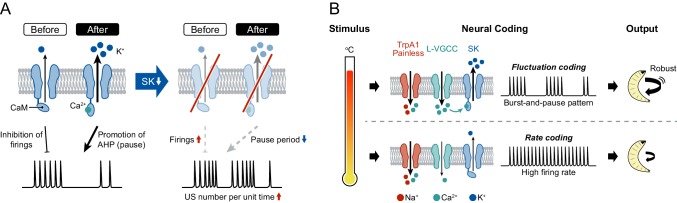
A model of information processing. (**A**) Two states of SK channels, and the activation level. Here, calmodulin (CaM, ellipses) is illustrated by tethering to the intracellular terminus of the SK channels. Before USs (or without USs), the small SK current inhibits the incidence of firings during burst periods. After USs, the large SK current promotes after-hyperpolarization (AHP), which induces pause periods. See further explanation in Discussion. (**B**) The regulatory mechanism of burst-and-pause firing patterns by functional coordination between L-type VGCCs and SK channels in Class IV neurons. General rate coding occurs in Class IV neurons at relatively low temperatures; this is mediated by thermoTRPs (TrpA1 and Painless) and many types of voltage-gated ion channels (not illustrated here). At higher temperature, however, L-type VGCCs and SK channels convert the firing from continuous high-frequency patterns into burst-and-pause patterns. This mechanism allows fluctuation coding in sensory neurons.

Although the increased number of USs in *SK* knockdown neurons may initially seem counterintuitive, it can be explained comprehensively by two states of SK channels, at low and high activation levels (Figure 4A): (i) Before USs occur, most SK channels are in the steady state because the Ca^2+^/calmodulin association is restricted at low [Ca^2+^]_i_, and the SK current slightly inhibits the incidence of firings during burst periods. Therefore, *SK* knockdown attenuates the inhibition of firings, which raises the occurrence rate of USs. (ii) In contrast, after USs occur with dendritic Ca^2+^ transients, the channels are shifted to the activation state by high [Ca^2+^]_i_, and the current greatly promotes after-hyperpolarization, which generates the pause periods. Thus, the knockdown dramatically decreases the pause periods, which shortens the time requiring one burst-and-pause firing pattern. Due to the two impacts on firings, the US number per unit time would be expected to increase upon *SK* knockdown.

**Figure 4—figure supplement 1.**
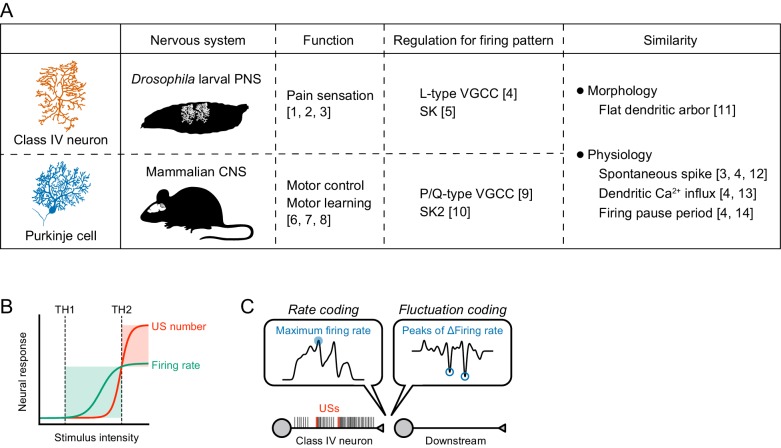
Information processing by the burst-and-pause firing pattern. (**A**) Comparison between Class IV neurons and Purkinje cells. These two neuronal types have similar profiles in both morphology and physiology. We suggest that the regulatory mechanism of the former burst-and-pause firing pattern is similar to that of the latter complex spike. The image of a Purkinje cell was modified from Hong and De Schutter (2008). References: [1] Hwang et al. (2007), [2] Tracey et al., 2003, [3] Xiang et al. (2010), [4] Terada et al., 2016, [5] This study, [6] Nguyen-Vu et al. (2013), [7] Porras-García et al. (2013), [8] Vogel et al. (2007), [9] Davie et al., 2008, [10] Grasselli et al. (2016), [11] Grueber et al. (2002), [12] Raman and Bean (1999), [13] Lev-Ram et al., 1992, and [14] Llinás and Sugimori (1980). (**B**) Sensory transduction model in Class IV neurons. The firing rate increases with stimulus intensity above the threshold of activation in neurons (TH1), but it saturates at much higher intensity. In contrast, the US number increases above the threshold, thereby inducing dendritic Ca^2+^ transients (TH2). (**C**) Reading model for burst-and-pause firing patterns in downstream neurons. Our present and previous study (Terada et al., 2016) suggest that the US number should be read out as peaks of firing rate fluctuations downstream of Class IV neurons, and that fluctuation coding occurs in the sensory nervous system. When USs occur, the maximum firing rate also tends to increase, and several time points of the maximum firing rate overlap with US timings (Figure 1—figure supplement 1B, Figure 3—figure supplement 1). These results suggest that the USs could still be read out in accordance with rate coding. However, L-type VGCC knockdown did not change the maximum firing rate (Figure 2—figure supplement 2B) but delayed avoidance behavior (Terada et al., 2016), which demonstrates that the rate coding itself cannot explain our findings, and supports our view that fluctuation coding occurs in Class IV neurons.

We hypothesize that the burst-and-pause firing patterns in Class IV neurons are regulated by functional coordination between L-type VGCCs and SK channels as follows (Figure 4B): (1) Thermosensitive channels including dTrpA1 and Painless (Luo et al., 2017; Neely et al., 2011; Tracey et al., 2003; Zhong et al., 2012) are activated by high-temperature stimulation and elicit the initial membrane depolarization in the dendritic arbors. (2) Once the membrane potential of soma exceeds a certain threshold by the prolonged stimulation, the neurons evoke action potentials and then increase firing rates with the intensity of stimulation (‘rate coding’). (3) When L-type VGCCs in the dendritic arbors are activated by the high-order depolarization, they induce a large Ca^2+^ influx, which rapidly activates SK channels. (4) The activated SK channels produce a hyperpolarizing current, thereby generating the pause periods (‘fluctuation coding’). We also suggest that other K^+^ channels may slightly contribute to the generation of pauses, because the pause periods were not completely abolished in *SK* knockdown neurons (Figure 3E, Figure 3—figure supplement 2D). Although the other candidate channels, such as *Sh* and *Shal*, are not activated by the [Ca^2+^]_i_ rise, most of them are voltage-dependent and hence hyperpolarize the membrane potential to some degree after depolarization, regardless of dendritic Ca^2+^ influx. Because the hyperpolarization suppresses the probability of firing, including US, the knockdown of those channels should lead to the increment of the US number.

In the mammalian cerebellar cortex, climbing fiber inputs evoke complex spikes of Purkinje cells, which induce a dendritic Ca^2+^ influx through Ca^2+^ spikes and subsequent pauses (Davie et al., 2008; Kitamura and Häusser, 2011; Llinás and Sugimori, 1980; Mathews et al., 2012). The pause periods of post-complex spikes are regulated by dendritic Ca^2+^ spikes, which are dependent on P/Q-type VGCCs (Davie et al., 2008), and are modulated by after-hyperpolarization, which is largely dependent on SK2 channels (Grasselli et al., 2016). Considering these observations, the regulatory mechanism of complex spikes is remarkably similar to that of burst-and-pause firing patterns in Class IV neurons (Figure 4—figure supplement 1A).

In principle, sensory neurons convert the intensity of stimuli into the magnitude of firing rates (Adrian, 1926). This form of rate coding also occurs in Class IV neurons at relatively low temperatures, and it is mediated by thermosensitive channels and many types of voltage-gated ion channels (Figure 4B, Figure 4—figure supplement 1B and C). At higher temperature, however, L-type VGCCs and SK channels modulate the firing, transitioning from continuous high-frequency patterns into burst-and-pause patterns. Thus, we propose that the firing-rate-fluctuation coding allows sensory neurons to transmit strong stimuli not covered in rate coding, thereby provoking robust avoidance behavior.

## Materials and methods

### *Drosophila* mutant and transgenic strains

The transgenic line expressing the FRET-based Ca^2+^ indicator TN-XXL (Mank et al., 2008) in Class IV neurons was *3×[ppk-TN-XXL] (attP40)* from our previous work (Terada et al., 2016). Mutants of one of the Anoctamin family channels, Subdued, were *subdued*^Δ5265^ and *Df(3R)Exel6184*, from C. Kim. A transgenic line expressing the split Gal4 was *R72F11_AD; R52F07_DBD* from T. Ohyama. A transgenic line expressing the FRET-based Cl^−^ indicator SuperClomeleon was *UAS-SuperClomeleon* (FBst0059847; Haynes et al., 2015) from the Bloomington Stock Center. Transgenic lines expressing channel RNAi were from the Bloomington Stock Center and Vienna *Drosophila* Resource Center (see also Figure 2—source data 1). Other transgenic lines were *pickpocket* (*ppk*)*-Gal4* (FBst0032078), *20 × UAS-GCaMP5G* (FBst0042037), *UAS-Dcr-2* (FBst0024651), *TrpA1-QF* (FBst0036348), *R69F06-Gal4* (FBst0039497), *10XQUAS-ChR2.T159C-HA* (FBst0052259), and *20XUAS-IVS-NES-jRCaMP1b-p10* (FBst0063793), from the Bloomington Stock Center.

Exact genotypes of individual animals used in figures are described below:

### Figure 1

*ppk-Gal4*/*20 × UAS-GCaMP5G (attP40)*

### Figure 2

(B–C) *ppk-Gal4*/*20 × UAS SuperClomeleon (attP40)*

(D–F) *ppk-Gal4, 3×[ppk-TN-XXL] (attP2)*, *UAS-each channel RNAi* (see Figure 2—source data 1)

### Figure 3

*w*^1118^; *ppk-Gal4*/+; *3×[ppk-TN-XXL] (attP2)*/+ (‘*ppk-Gal4*’)

*UAS-SK RNAi*^HMJ21196^/+; *3×[ppk-TN-XXL] (attP2)*/+ (‘*UAS-SK RNAi*’)

*ppk-Gal4*/*UAS-SK RNAi*^HMJ21196^; *3×[ppk-TN-XXL] (attP2)*/+ (‘*ppk>SK RNAi*’)

### Figure 1—figure supplement 1

(B) *ppk-Gal4*/*20 × UAS-GCaMP5G (attP40)*

### Figure 2—figure supplement 1

(A–F) *3×[ppk-TN-XXL] (attP40)*/+ (‘WT’)

*3×[ppk-TN-XXL] (attP40)*/+; *subdued*^Δ5265^/*Df(3R)Exel6184* (‘*subdued*’)

(G–H) *ppk-Gal4*/*20 × UAS SuperClomeleon (attP40); subdued*^Δ5265^/*Df(3R)Exel6184*

### Figure 2—figure supplement 2

*ppk-Gal4, 3×[ppk-TN-XXL] (attP2)*, *UAS-each channel RNAi* (see Figure 2—source data 1)

### Figure 3—figure supplement 2

*w*^1118^; *ppk-Gal4*/+; *UAS-Dcr-2/+* (‘Control’)

*w*^1118^; *ppk-Gal4*/+; *UAS-Dcr-2/UAS-SK RNAi*^GD12601^ (‘*SK RNAi*^GD^’)

*w*^1118^; *ppk-Gal4*/*UAS-SK RNAi*^KK107699^; *UAS-Dcr-2*/+ (‘*SK RNAi*^KK^’)

### Figure 3—figure supplement 1

(A) *w*^1118^; *ppk-Gal4*/+; *3×[ppk-TN-XXL] (attP2)*/+ (‘*ppk-Gal4*’)

*UAS-SK RNAi*^HMJ21196^/+; *3×[ppk-TN-XXL] (attP2)*/+ (‘*UAS-SK RNAi*^HMJ^’)

*ppk-Gal4*/*UAS-SK RNAi*^HMJ21196^; *3×[ppk-TN-XXL] (attP2)*/+ (‘*ppk>SK RNAi*^HMJ^’)

(B)*w*^1118^; *ppk-Gal4*/+; *UAS-Dcr-2/+* (‘Control’)

*w*^1118^; *ppk-Gal4*/+; *UAS-Dcr-2/UAS-SK RNAi*^GD12601^ (‘*ppk>SK RNAi*^GD^’)

*w*^1118^; *ppk-Gal4*/*UAS-SK RNAi*^KK107699^; *UAS-Dcr-2*/+ (‘*ppk>SK RNAi*^KK^’)

### Figure 3—figure supplement 3

*w*^1118^; *ppk-Gal4*/+; *3×[ppk-TN-XXL] (attP2)*/+ (‘*ppk-Gal4*’)

*UAS-SK RNAi*^HMJ21196^/+; *3×[ppk-TN-XXL] (attP2)*/+ (‘*UAS-SK RNAi*’)

*ppk-Gal4*/*UAS-SK RNAi*^HMJ21196^; *3×[ppk-TN-XXL] (attP2)*/+ (‘*ppk>SK RNAi*’)

*3×[ppk-TN-XXL] (attP2)*/*UAS-Sur RNAi*^GL00506^

*ppk-Gal4*/+; *3×[ppk-TN-XXL] (attP2)*/*UAS-Sur RNAi*^GL00506^

### Figure 3—figure supplement 4

*w*^1118^; *ppk-Gal4*/+; *3×[ppk-TN-XXL] (attP2)*/+ (‘*ppk-Gal4*’)

*ppk-Gal4, 3×[ppk-TN-XXL] (attP2)*, *UAS-each channel RNAi* (see Figure 2—source data 1)

### Figure 3—figure supplement 5

(B–F)

*TrpA1-QF 10XQUAS-ChR2.T159C-HA*/*R72F11_AD*; *20XUAS-IVS-NES-jRCaMP1b-p10*/*R52F07_AD*

(G–H)

*TrpA1-QF 10XQUAS-ChR2.T159C-HA*/+; *20XUAS-IVS-NES-jRCaMP1b-p10*/*R69F06-Gal4*

(‘*Class IV>ChR2*, *Goro>jRCaMP*’)

### Electrophysiology and IR-laser irradiation

Preparation of larvae and extracellular recording were performed as previously described (Terada et al., 2016, Figure 1—figure supplement 1A). The foci of the infrared (IR)-laser irradiation were targeted onto the proximal dendritic arbors, essentially as described in our previous analyses. The time window for the experimental irradiation was 1 s except for data for Figure 2—figure supplement 1C (30 mW IR, 5 s) and Figure 3A (36-mW IR, 5 s), which were quantified during the initial 1 s. Quantification of the maximum firing rate and the peak number of firing rate fluctuations was performed as previously described (Terada et al., 2016) with slight modifications (see Figure 2—figure supplement 2A). For quantification of the pause period, we excluded USs that had occurred around the shutdown of IR-laser irradiation and were not accompanied by additional spikes during the irradiation.

### Ca^2+^ imaging

We used a TN-XXL indicator except for Figure 1 because it allowed more robust quantitative analysis in the presence of perturbations along Z-axis motions by larval body wall muscles. Ca^2+^ imaging of TN-XXL-expressing Class IV neurons was performed as previously described (Terada et al., 2016). ΔR is the change of fluorescence ratio and ΔR_peak_ is defined as the maximum. Ca^2+^ imaging of GCaMP5G was performed on filet preparations. GCaMP5G was excited with a 445 nm diode laser (CUBE 445–40C, Coherent, Santa Clara, CA). Images were acquired at 128 × 128 pixels with 1 × 1 binning, in a 14-bit dynamic range, and with 10 ms exposure time. The fluorescence signal was captured by the imagers with 100 Hz through 578/105 bandpass filters (Semrock, Lake Forest, IL). The fluorescence change was defined as:

$$\Delta F/F_{0}=\left(F_{n}-F_{0}\right)/F_{0}$$

where F_n_ is the fluorescence at time point n, and F_0_ is the average fluorescence before starting IR-laser irradiation (time window 100 ms). F_peak_ is defined as the maximum amplitude of ΔF/F_0_. The fluorescence itself declined during IR-laser irradiation, so the decay was subtracted for quantification.

### Estimation of the onset timing of Ca^2+^ transients by curve fitting

Estimation of onset timings of Ca^2+^ transients was performed as previously described (Grewe et al., 2010; Lütcke et al., 2013) with slight modifications:

$$f_{\text{Ca}}\left(t\right)=A\left(1-e^{-\left(t-t_{0}\right)/\tau_{\text{on}}}\right)\cdot e^{-\left(t-t_{0}\right)/\tau_{\text{off}}\;},\text{ for }t>t_{0}$$

$$f_{\text{Ca}}\left(t\right)=0,\text{for}t\leq t_{0}$$

Here, $t_{0}$ denotes the onset of Ca^2+^ transients, $\tau_{\text{on}}$ the onset rise time constant, $\tau_{\text{off}}$ the decay time constant, and A an amplitude scale parameter.$\tau_{\text{on}}$ and $\tau_{\text{off}}$ were manually adjusted for precise curve fitting in each trial ($\tau_{\text{on}}$=50–500 ms, $\tau_{\text{off}}$ = 1.5–10 s). The value of A is dependent on the maximum of each Ca^2+^ transient. Before fitting, a baseline offset was subtracted from the trace segment. We used MATLAB scripts provided in Lütcke et al. (2013).

### Cl^−^ imaging

Cl^−^ imaging of SuperClomeleon-expressing Class IV neurons was performed on whole-mount preparations. The data acquisition system was the same as for Ca^2+^ imaging of TN-XXL (Terada et al., 2016). The ratio of SuperClomeleon was defined as:

$$Ratio_{\text{SuperClomeleon}}=\left(YFP_{\text{unmasked}}-YFP_{\text{masked}}\right)/\left(CFP_{\text{unmasked}}-CFP_{\text{masked}}\right)$$

where YFP_unmasked_ and CFP_unmasked_ are signals of outlined cellular regions, and YFP_masked_ and CFP_masked_ are those of background.

### Thermal behavioral assay

Animals were raised at 25˚C in an incubator with 12 hr light/dark cycles, and humidity was manually controlled (75–80%). Wandering third-instar larvae were gently picked up from the vial, washed three times with deionized water, and transferred to a 140 × 100 mm petri dish with fresh 2% agarose. Excessive water was removed from the animals. For acclimation, animals were allowed to rest on the plate for at least 5 min before testing. The response latency was measured as the time interval from the point at which the larva was first contacted by the probe until it initiated the first 360˚ rotation. The time window was 5 s when we could maintain the contact more precisely than the general time window (10 s). About 20 larvae in the control and experimental groups were tested on the same day, and the assays were repeated for several days.

### Image acquisition and quantification of dendritic morphology

Imaging ddaC neurons in whole-mount larvae was done as previously described (Shimono et al., 2014), with slight modifications. Wandering third-instar larvae were gently picked up from the vial, and washed once with 0.7% NaCl and 0.3% Triton X-100, and three times with deionized water. They were mounted in 50% glycerol on slides, between spacers made of vinyl tape. Images of YFP fluorescence in TN-XXL were acquired using a Nikon C1 laser-scanning confocal microscope. Original images of each neuron were composed of maximum intensity projections of confocal micrographs.

Dendritic coverage was quantified as previously described (Honjo et al., 2016) with appropriate modifications (Figure 3—figure supplement 3A). Original images were inverted with black and white, and the images were converted through a Laplacian filter to enhance the edge contrast and the VanderBrug operator (Vanderbrug, 1976; VanderBrug, 1977) to enhance the line contrast. The images were binarized to detect the enhanced parts. The images were converted through a maximum filter to interpolate between separated dendrites. Spotted noise was removed by labeling. The images were overlaid with a grid of 34 × 34 pixel squares (14 × 14 µm), and squares containing signals were counted to calculate the dendritic coverage score. The preceding quantification steps were automatically processed with a MATLAB script. After that, false-positives and false-negatives were manually corrected on a MATLAB application using a graphical user interface (GUI).

### Optogenetic neural activation

Optogenetic activation of Class IV neurons was performed as previously described (Terada et al., 2016). Larvae expressing the ChR2 variant in Class IV neurons driven by *TrpA1-QF (attP40)* (Petersen and Stowers, 2011; Yoshino et al., 2017) were grown on fly food containing all trans-retinal (R2500; Sigma-Aldrich) at a final concentration of 0.5 mM. For optogenetic activation, a single long pulse (continuous illumination) or multiple cycles of 100 ms pulses followed by 100 ms pause intervals (intermittent illumination) were applied by using a collimated LED light lamp (M470L3-C1; ThorLabs, Newton, NJ) with an emission peak at around 470 nm (0.37 mW/mm^2^). Each cell was stimulated by two illumination patterns temporally separated by a pause interval of at least 1.5 min. The first stimulus was a continuous pattern, and the second was an intermittent one in half of the trials; and the sequence of stimulus patterns was reversed in the other half. We did not find any differences of neuronal activities dependent on the order of the two types of illuminations.

Ca^2+^ imaging of jRCaMP-expressing Goro neurons was performed on optimized filet preparations as follows: (i) After basic preparations on a glass slide, anterior thoracic epidermis was cut off. (ii) Epidermis under the larval brain was slit vertically from the anterior side, and the brain was gently pinned on the slide. (iii) The samples were incubated with 7 mM monosodium glutamate (Nacalai, Kyoto, Japan) for 8 min to prevent muscle contractions and eliminate motor feedback to the sensory circuits by saturating glutamate receptors at the neuromuscular junction (Kaneko et al., 2017).

Imaging was done as previously described (Arata et al., 2017), with slight modifications. Data were collected on an IX71 microscope (Olympus) equipped with an objective (UPLSAPO60XS NA 1.3, Olympus), Nipkow disk confocal system (CSU10, Yokogawa Electric, Tokyo, Japan), and an EM-CCD camera (iXon^EM^+ DU-888, Andor Technology, Belfast, UK). jRCaMP was excited with a 561 nm diode laser (Sapphire, Coherent, Santa Clara, CA), and was captured by the imagers at 1.5 s intervals through 610/60 bandpass filters (Chroma Technology, Bellows Falls, VT). The above imaging system was controlled by MetaMorph software (Molecular Devices, Sunnyvale, CA). Each sample was stimulated once either by continuous illumination or by intermittent illumination. Quantification of the fluorescence change was the same as for Ca^2+^ imaging of GCaMP5G.

### Statistics

Data were analyzed and plotted using ImageJ (National Institutes of Health, Bethesda, MD), MATLAB (The MathWorks, Natick, MA), and Microsoft Excel (Microsoft Corporation, Redmond, WA). Details for each figure are shown in source data. To prevent misinterpretation as outliers in some figures (Figure 2E and F; Figure 2—figure supplement 2), p<0.05 and p<0.01 are indicated by double and triple asterisks, respectively.

### Abbreviations

[Ca^2+^]_i_, intracellular Ca^2+^ concentration; [Cl^−^]_i_, intracellular Cl^−^ concentration; GUI, graphical user interface; IR, infrared; ISI, interspike interval; K_2P_, Two-pore domain K^+^ channel; K_Ca_, Ca^2+^-activated K^+^ channel; K_ir_, Inward rectifier K^+^ channel; K_v_, voltage-gated K^+^ channel; NR, no response group; ns, not significant; *ppk*, *pickpocket*; SK channel, small conductance Ca^2+^-activated K^+^ channel; US, unconventional spike; VGCC, voltage-gated Ca^2+^ channel.

## Additional files

Transparent reporting form
